# Prognostic factors and outcome of pineoblastoma: 10 years single-center experience

**DOI:** 10.1186/s43046-021-00083-3

**Published:** 2021-09-20

**Authors:** Ahmed Elhemaly, Mohamed S. Zaghloul, Soha Ahmed, Hala Taha, Amal Refaat, Eslam Maher, Mohamed El-Beltagy, Wael Zekry

**Affiliations:** 1 Pediatric Oncology, National Cancer Institute (NCI), Cairo University and Children Cancer Hospital of Egypt (CCHE), Cairo, Egypt; 2 Department of Radiation Oncology, National Cancer Institute, Cairo University and Children Cancer Hospital (CCHE), Cairo, Egypt; 3 Clinical Oncology Department, Aswan University, Aswan, Egypt; 4 Department of Pathology, National Cancer Institute, Cairo University and Children Cancer Hospital (CCHE), Cairo, Egypt; 5 Radio-Diagnosis Department, National Cancer Institute & Children’s Cancer Hospital, Cairo, Egypt; 6 Clinical Research Department, CCHE (57357 hospital), Cairo, Egypt; 7 Department of Neurosurgery Children’s Cancer Hospital, Egypt and Faculty of Medicine Cairo University, Cairo, Egypt

**Keywords:** Pineoblastoma, Prognostic factors, Outcome

## Abstract

**Background:**

The survival of pineoblastoma patients is low, particularly in infants and those with metastatic disease. This study aimed to analyze the prognostic factors affecting the outcome of Pineoblastoma in different age groups.

**Methods:**

A retrospective study included 33 patients. Twenty-two patients older than 3 years had upfront surgery, followed by induction CSI then 6 cycles of chemotherapy.

Eleven patients younger than 3 years underwent surgery, followed by induction chemotherapy then radiation therapy. Focal irradiation (54 Gy) was administrated in six patients, and CSI (23.4 Gy) with booster dose 30.6 Gy to the tumor bed in two patients followed by 4 cycles of chemotherapy.

**Results:**

Patient’s age showed a significant impact on the outcome (*P* value = 0.001 for EFS and 0.002 for OS). The metastases’ presence did not impact the outcome negatively. The survival of patients with metastatic disease did not differ between age groups. However, age had a significant impact on the outcome of M0 disease, with 3-year EFS and OS of 65.3% and 74%, respectively, in the older group compared to 0% for both rates in younger patients. CSI showed a positive impact on survival. For all cases, the 3-year OS and EFS were 46.7% and 44.4%, respectively.

**Conclusions:**

A multimodality approach is needed to treat this aggressive disease. Inadequate dose intensity affected our patients’ outcome negatively. A more aggressive approach using high-dose chemotherapy or CSI may be required to improve infantile pineoblastoma’s dismal outcome. Focal radiotherapy is not an efficacious treatment in infants due to its high-metastatic potential. Molecular typing should be considered to label patients who need a more intensified approach.

## Background

Pineoblastomas (PBs) represent the most aggressive pineal parenchymal tumors.

Pineal parenchymal tumors are rare central nervous system (CNS) neoplasms with different histological appearance and clinical phenotypes. They range from World Health Organization (WHO) grade 1 pineocytomas, to WHO grade 2–3 pineal parenchymal tumors of intermediate diferentiation (PPTIDs), and grade 4 pineoblastomas (PBs), an embryonal tumor predominantly of pediatric onset. Resection alone is sufficient for treatment of pineocytoma, the optimal adjuvant therapy needed for patients with PPTIDs is still unclear [1].

Treatment consists of maximal resection and adjuvant chemotherapy/radiotherapy, resulting in a median survival of 20 months [2].The incidence is approximately 6 cases in 1000 patients per year [3, 4]. Symptoms result mainly from compression of the nearby structures, such as the tectum, resulting in ophthalmoplegia and obstructive hydrocephalus [5, 6]. Successful management requires multimodal therapies, such as surgery, chemotherapy (CTH), and radiotherapy (RTH), ± high-dose chemotherapy (HDCTH), followed by stem-cell rescue [3].The impact of the extent of the resection on the outcome is a matter of debate; some believe that aggressive surgery is needed to improve the outcome, but others do not [7]. Recently, Liu et al. conducted a prospective multicenter SJMB03 and SJYC07 trial about pineoblastoma; they concluded that gross total resection (GTR) was significantly associated with superior outcomes for patients more than 3 years of age [8]. HDCTH in pineoblastoma is increasingly used with or without the application of focal or craniospinal (CSI) irradiation [9]. The survival of pineoblastoma patients is low compared to medulloblastoma, particularly in infants and in patients with metastatic disease [10]. This study aimed to analyze different prognostic factors affecting intracranial pineoblastoma outcomes in our center’s 10 years of experience.

## Methods

Following institutional review board approval, we retrospectively identified 33 pediatric patients (≤ 18 years old) diagnosed with pineoblastoma treated from January 2008 to December 2017. These were all available patients with pineoblastoma in our center. Patients with trilateral retinoblastoma were excluded from the study due to their incomplete data and different treatment protocol applied. All patients did tumor markers upfront; to exclude intracranial non-germinomatous germ cell tumor (NGGCT), those with low markers did endoscopic biopsy. Relieving the elevated intracranial pressure was done by ventriculoperitoneal (V/P) shunt or endoscopic ventriculostomy. Patients’ records were reviewed electronically for different prognostic factors which may have affected the outcome, such as the patient’s age, presence of metastases (M + ve), the extent of resection, adjuvant therapy administration (chemotherapy, radiotherapy, or both), radiotherapy field (focal versus CSI), and radiological response post-therapy. These factors were correlated with overall survival (OS) and event-free survival (EFS). All patients had documented pathology without molecular subgrouping, which is not available in our center.

The authors state that they have obtained appropriate institutional review board approval and have followed the principles outlined in the Declaration of Helsinki. Besides, informed consent has been obtained from the participants involved.

### Staging of the patients

All patients did upfront craniospinal MRI and craniospinal fluid (CSF) cytology to detect any evidence of metastases. Metastatic disease included the presence of positive CSF cytology and intracranial or intra-spinal seedling or metastases.

### Definitions

The extent of resection was defined as follows: GTR (gross total)/NTR (near-total resection): > 90% resection of the tumor; STR (subtotal resection): > 50% and < 90% resection of the tumor; and biopsy: < 50% resection [10]. The radiological response post-induction was categorized into complete response (CR): no evidence of the tumor; partial response (PR): 50% reduction in tumor size (three-dimensional calculation); minimal response (MR): 25 to 50% reduction in tumor size; stable disease (SD): less than 25% decrease in tumor size; and progressive disease (PD): 25% increase in tumor size or appearance of new lesions [9].

### Treatment

#### Patients older than 3 years of age

Upfront surgery was performed, followed by induction CSI. The fractionated CSI dose was 36.0 Gy (for M0 and M + ve disease) with a boost to the tumor bed (30.6 Gy) to reach 54–55.8 Gy total tumor bed dose, according to COG ACNS0332 protocol. Weekly, vincristine was given concomitantly with radiotherapy. Six cycles of maintenance chemotherapy (cisplatin, cyclophosphamide, and vincristine) were given post-RTH. MRIs of the brain and spine were done post-induction, and every 3 cycles of maintenance. All patients received adjuvant chemotherapy and radiotherapy, except for two patients who died from progressive metastatic disease shortly after diagnosis due to their guardians' refusals to give any adjuvant therapy.

#### Patients younger than 3 years of age

Maximum safe resections were performed before induction chemotherapy, according to the COGP9934 protocol. This policy aimed to delay radiotherapy exposure for the fear of the associated endocrinological, intellectual, and memory changes. Six patients received focal irradiation (54 Gy) to the tumor bed. According to the tumor board decision, two patients (one with M + ve and the other with M0 disease) received CSI (23.4 Gy) followed by a boost dose up to 54 Gy to the tumor bed. Four cycles of maintenance chemotherapy (cyclophosphamide, etoposide, and vincristine) were given post-RTH. Radiological assessments were done post-induction, post-radiation therapy, and at the end of treatment. HDCTH was not a part of the treatment protocol in any age group. Radiological responses post-induction RTH and CTH in both age groups were analyzed and correlated with the outcome.

## Statistical analysis

Statistical analysis was done using IBM© SPSS© Statistics version 22. Numerical data were expressed as median and range. Qualitative data were expressed as frequency and percentage. Survival analysis was done using the Kaplan-Meier method, and a comparison between two survival curves was made using the log-rank test. A *p* value < 0.05 was considered significant. Event-free survival (EFS) was measured from the date of diagnosis to progression, relapse, or death. Overall survival (OS) was calculated from the date of diagnosis until the date of death or the last follow-up. We calculated the minimum sample size required for a multivariable proportional hazards model using three prognostic factors with four parameters (age group, the extent of resection, and presence of metastasis) per the criteria proposed by Riley et al. to avoid over fitting [11]. Assuming adjusted Cox-Snell *R*^2^ = 0.54 as derived from the c-statistic provided by a previous SEER analysis of pineoblastoma [12]. A minimum sample size of 70 patients is needed. Our cohort was not powered for such an analysis.

## Results

The study included 33 patients (15 males, 18 females). Patients' ages ranged from 1.5 to 17 years (median 4.7 years). Twenty-two patients were > 3 years old at diagnosis (13 M0, 9 M + ve). Twenty (20/22) (13 M0, 7 M + ve) patients received 36 Gy fractionated CSI. Eleven patients were < 3 years of age (4 M0, 7 M + ve). Six (6/11) patients received focal RTH (3 M0, 3 M + ve). Two (2/11) patients received fractioned CSI (1 M0, 1 M + ve). Sixteen (16/33) (48.4%) patients were metastatic initially. All included patients were compliant to the radiotherapy and the chemotherapy cycles with no significant reported toxicity. Regarding surgical intervention, 25 patients (75.8%) underwent biopsy, 6 patients achieved GTR/NTR, and 2 patients did STR (Table 1).

**Table 1:** Baseline characteristics of the studied patients

	Variable	Number (33 patients) (%)
• **Age**	Less than 3	11(33.3)
	More than 3	22(66.7)
• **Gender**	Male	15(45.5)
	Female	18(54.5)
• **Metastatic state**	Non metastatic	17 (51.5)
	Metastatic	16(48.5)
• **Initial extent of resection**	GTR/NTR	6(18.2)
	Subtotal	2(6.1)
	Biopsy	25(75.8)
• **Adjuvant chemotherapy**	Yes	30 (90.9)
	No	3(9.1 )
• **Adjuvant radiotherapy**	Yes	28(84.8)
	No	5(15.2)
• **Type of radiotherapy**	Focal	6(18.2)
	CSI	22(66.7)
• **Radiological response post therapy**	CR	11(33.3)
	PR	12(36.3)
	MR	4(12.1)
	SD	1(3)
	PD	2(6)
	ND	3(9)

*GTR gross total resection, *NTR* near total resection, *CSI* craniospinal irradiation, *CR* complete response, *PR* partial response, *MR* minimal response, *SD* stationary disease, *PD* progressive disease, *N.D* not done

### Prognostic factors

**Fig. 1:**
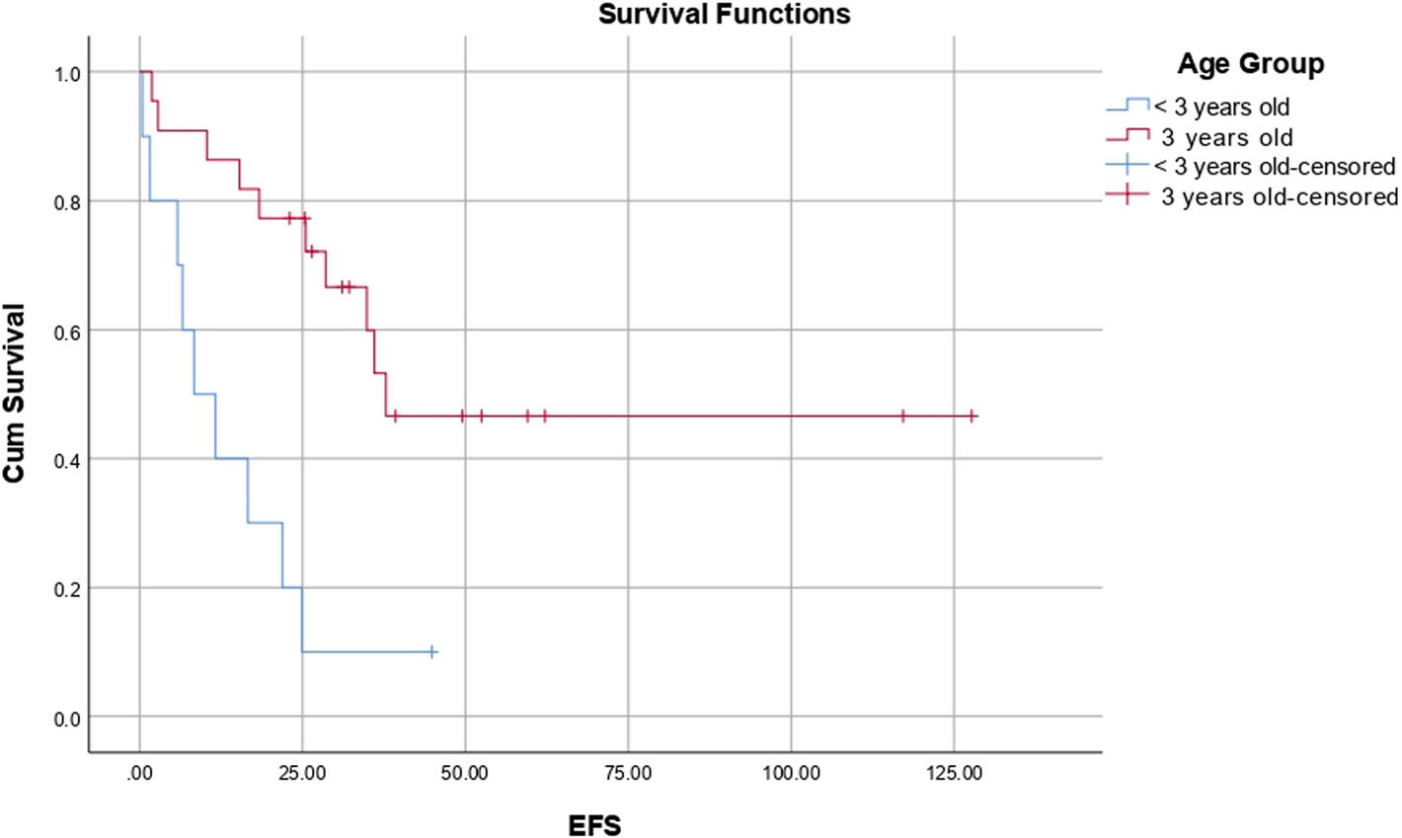
Correlation between patients’ ages and event-free survival

**Fig. 2:**
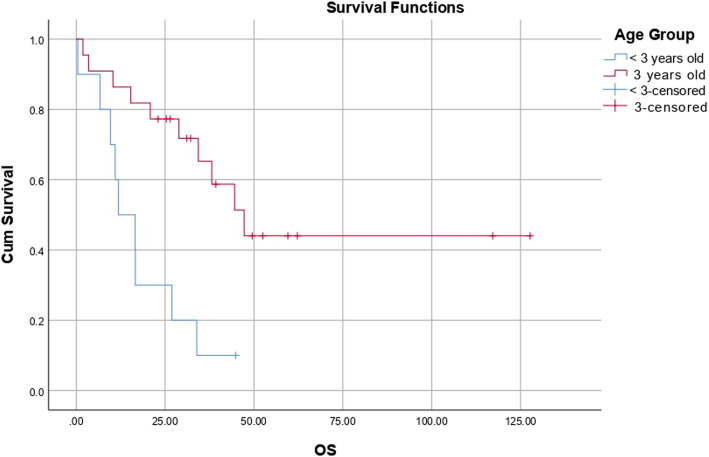
Correlation between patients’ ages and overall survival

**Fig. 3:**
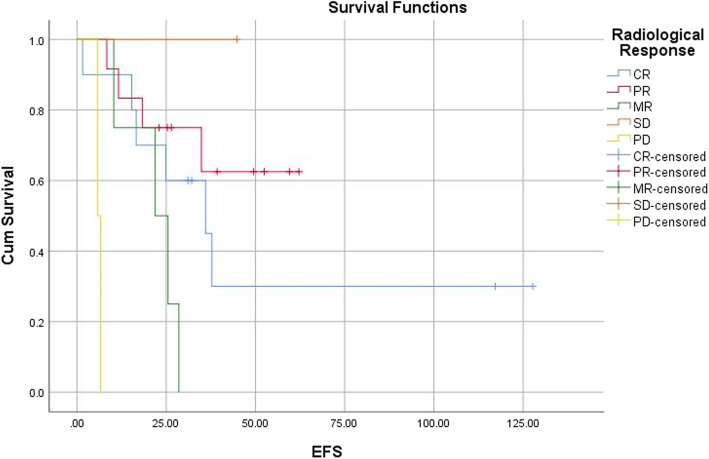
Correlation between radiological response post-induction and event-free survival

**Fig. 4:**
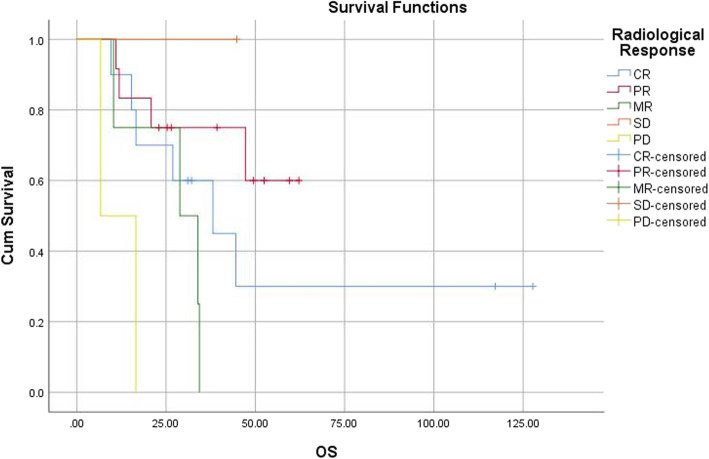
Correlation between radiological response post-induction and overall survival

Patient’s age showed a significant impact on the outcome, with 3-year EFS and OS of 10% (95% confidence interval [CI] 8.6–28.6) for the age group under 3 years compared to the 3-year EFS of 59.9% (95% CI 37.8–82) and OS of 65.2% (95% CI 43.8–86.6) for the older age group (*P* value = 0.001 for EFS and 0.002 for OS) (Figs. 1 and 2). The metastases’ presence (M +ve) did not significantly impact the outcome negatively (*P* value = 0.16 for EFS and 0.17 for OS). The survival of patients with metastatic disease was not significantly different between age groups (*P* value = 0.13 for EFS and 0.2 for OS). However, age had a significant impact on the outcome of M0 disease, with 3-year EFS and OS of 65.3% (95% CI 13.5–86.5) and 74% (95% CI 48.1–99.9), respectively, in the older group compared to 0% for both rates in the younger age group (*P* value = 0.001 for EFS and 0.002 for OS). The extent of resection did not show a significant impact on the outcome in both age groups. but this is not conclusive due to small number of patients who did GTR. Three (3/33) patients did not receive adjuvant chemotherapy. Receiving adjuvant chemotherapy had a significant positive impact on survival, with 3-year EFS and OS of 49% (95% CI 43.8–86.6) and 51.5% (95% CI 31.9–71.1), respectively, for the chemotherapy group compared to 0% for both in the non-chemotherapy group (*P* value < 0.001 for both EFS and OS). Radiotherapy administration significantly improved the outcome, with 3-year EFS and OS of 50.7% (95% CI 31.1–70.3) and 53.3% (95% CI 33.5–73.1), respectively. On the contrary, all patients who did not receive radiotherapy died (*P* value < 0.001 for both EFS and OS). Moreover, the field of RTH had a significant impact on survival. CSI was associated with better outcome compared to focal irradiation, with 3-year EFS and OS of 59.9% (95% CI 37.8–82) and 65.2% (95% CI 43.8–86.6) for CSI compared to 3-year EFS and OS of 16.7% (95% CI 0–46.5) for focal irradiation (*P* value = 0.02 for EFS, 0.04 for OS). Objective radiological response (CR and PR) was observed in 18 (18/22) (78.2%) older patients and 5 (5/11) (45.4%) younger patients. Radiological response at the end of induction showed a significant positive impact on survival (*P* value < 0.001 for EFS and 0.004 for OS) (shown in Figs. 3 and 4). All correlations are summarized in Table 2.

**Table 2:** Correlation between different prognostic factors and survival

	OS at 36 months (%)	95% CI	***P*** value	EFS at 36 months (%)	95% CI	***p*** value
**Age**						
**< 3 years**	**10**	8.6–28.6	**0.002**	**10**	8.6–28.6	**0.001**
**> 3 years**	**65.2**	43.8–86.6		**59.9**	37.8–82	
**Metastatic disease**						
**M0**	**54.5**	28.8–80.2	**0.16**	**49.9**	24–75.2	**0.17**
**M + ve**	**37.3**	11.2–63.4		**37.3**	11.2–63.4	
**Metastatic state in each age group:**						
**< 3 years**	**16.7**	0–46.2	**0.2**	**16.7**	0–46.2	**0.13**
**> 3 years**	**50**	13–86.5		**50**	13.5–86.5	
**M0 in each group**						
**< 3 years**	**0**	**NA**	**0.002**	**0**	**NA**	**0.001**
**> 3 years**	**74**	48.1–99.9		**65.3**	13.5–86.5	
**Extent of resection:**						
**GTR/NTR**	**50**	**10–90**	**0.62**	**50**	**10–90**	**0.71**
**STR**	**0**	**NA**		**0**	**NA**	
**Biopsy**	**50**	**28–72**		**46.8**	25.2–68.4	
**Extent of surgery**						
**Less than 3**						
**GTR/NTR**	**0**	**NA**	**0.92**	**0**	**NA**	**0.87**
**Biopsy**	**14.3**	0–40.2		**14.3**	**0–40.2**	
**More than 3**						
**GTR/NTR**	**100**	**NA**	**0.11**	**100**	**NA**	**0.17**
**STR**	**0**	**NA**		**0**	**NA**	
**Biopsy**	**66.9**	42–91.8		**59.6**	33.1–86.1	
**Radiotherapy:**						
**Yes**	**53.3**	33.5–73.1	**< 0.001**	**50.7**	31.1–70.3	**< 0.001**
**No**	**0**	NA		**0**	NA	
**Radiotherapy field:**						
CSI	65.2	43.8–86.6	0.04	59.9	37.8–82	0.02
Focal	16.7	0–46.5		16.7	0–46.5	
**Chemotherapy:**						
Yes	51.5	31.9–71.1		49	43.8–86.6	
No	0	NA	< 0.001	0	NA	< 0.001
**Radiological response**						
CR	75	29.6–90.4		62.5	29.6–90.4	
PR	60	50.5–99.5		60	32.1–92.9	
MR	0	NA	0.004	0	NA	< 0.001
SD	0	NA		0	NA	
PD	0	NA		0	NA	

### Outcome of metastatic disease

Sixteen patients showed initial metastatic disease. In the younger age group (7/11), three patients received focal RTH, one received CSI, two progressed after the induction chemotherapy before receiving RTH, and one died immediately postoperatively. Six (6/7) (85.7%) patients showed distant progression and died. The only surviving patient is the one who received CSI. Of older patients, seven (7/9) patients received CSI and adjuvant chemotherapy, and two (2/9) died from the progressive metastatic disease without starting any adjuvant therapy. Four (4/9) (44.4%) patients died from progressive metastatic disease post-therapy. Three (3/9) (30%) are still alive without evidence of disease progression.

### Survival outcome

One patient died from surgical complications before any adjuvant therapy. Nineteen (19/33) (57.5%) patients (10 from the younger age group, 9 from the older age group) developed disease progression post-therapy and died. Ten (10/19) patients were initially metastatic. Most of the failures occurred distally (15 patients showed disseminated disease; four patients developed local progression). Ten (10/11; 90.9%) patients of the younger group progressed and died. One died postoperatively, two died from progressive metastatic disease post-induction chemotherapy, and the remaining patients showed metastatic progression during other therapy phases. Seven (7/10) patients were initially metastatic, and six (6/7) patients showed spinal progression. All patients received focal radiotherapy. All M0 patients in this age group died. The only surviving patient in the younger age group initially had metastatic disease and received CSI. The median follow-up period was 44.8 months. The studied patients’ median OS and EFS were 34.3 months (95% CI 20.96–47.72) and 28.55 months (95% CI 13.45–43.65), respectively. No RTH/CTH-related mortality occurred. For all cases, the 3-year OS and EFS were 46.7% (95% CI 28.3–65.1) and 44.4% (95% CI 26.4–62.4), respectively.

## Discussion

Pineoblastoma is a rare, embryonic tumor with sparse data in literature about the outcome and prognostic factors affecting the survival. Some studies support that patients with pineoblastomas have a worse outcome compared to other primitive neuroectodermal tumors (sPNETs) [13]. In this study, younger patients had significantly low EFS and OS. The impact of age on outcome is mostly due to different molecular subgroups that affected the response to treatment and metastases occurrence. Recent studies proved that there are four molecular subgroups in pineoblastoma (A, B, B-like, and FOXR2) exist. Group A occurs in infants, and B and B-like groups occur in older age with 50% and 0% incidence of metastases, respectively [8]. The COG ACNS0332 trial reported a 5-year EFS of 62.8% in pineoblastoma patients older than 3 years, which is better than our results [14]. A pooled analysis from 11 centers under the umbrella of SIOP reported 5-year PFS rates of 63% and 11% for patients older and younger than 4 years of age, respectively [15].

The presence of the metastatic disease did not affect survival in our study. Gururangan S et al. reported a surprisingly higher survival rate of 75% for metastatic and localized diseases. These unexpected results may be attributed to high-dose chemotherapy administration, which nullified the negative impact of metastatic disease [9]. The SIOP multicentric work proved that metastatic disease was an independent adverse prognostic factor, and using HDCTH did not improve survival [15]. Contrarily, Jakacki RI et al. reported that metastatic disease had no significant impact on survival [16]. In our study, there was no significant impact of age on the outcome of metastatic disease. However, it was apparent that young patients with metastatic disease showed a much lower cure rate, where 85% of the younger patients died from progressive disease compared to 56% in the older age group. In our study, the 3-year OS of older patients with M + ve disease was 50% compared to 5-year OS of 60.3% in the SJMB03 trial. The SJYC07 trial reported a 2-year OS of 0% for infantile patients with M + ve disease, comparable with our results (10% survival) [8]. The common element between our study and the SJYC07 trial is that neither used mega therapy in infants. In this study, the extent of resection did not affect the outcome as in the SIOP data but this is not conclusive in our study due to small number of patients who underwent GTR [15]. Low number of gross total resection was due to reluctance of the neurosurgeon to pursue this risky procedure with no clear evidence of the impact of GTR on the outcome.

However, St. Jude’s report concluded that GTR was significantly associated with a better outcome for patients over 3 years (PFS, *P* = 0.005, OS, *P* = 0.008) with no impact on the younger age group [8]. Another St. Jude report concluded that there was no outcome difference between GTR and STR, but when controlling for age, 80% of the GTR group and 50% of the STR/biopsy group were alive without evidence of progression [10]. Adjuvant therapy was a significant predictor of the outcome in our study. All patients who did not receive adjuvant CTH/RTH (due to guardians’ refusal), died. This emphasizes that pineoblastoma is an aggressive disease that cannot be treated by surgery alone. CSI showed a more positive impact on survival than focal irradiation, taking in consideration that all older patients received CSI while most of the younger patients received focal RTH. The better results with CSI may be due to the impact of the age group and the related molecular subtypes.

Patients younger than 3 years of age were assigned for focal radiotherapy for fear of developmental, intellectual and toxicity which may be encountered by CSI. Patients close to or older than 3 years of age were assigned to CSI as they can better tolerate CSI with less side effects aiming to improve the outcome.

Mynarek M et al. concluded that radiation therapy was the most important prognostic factor where most of the patients received CSI. Five non-metastatic young patients achieved cure with focal radiotherapy and HDCTH [15]. Focal RTH delivery may explain young patients’ unfortunate outcomes in our study (even the non-metastatic ones), as they were offered focal RTH without mega therapy. In this study, most of the failures (15/19) occurred distally, regardless of their metastatic state upfront. These results pointed out that focal RTH is not efficient in treating pineoblastoma, especially in infants who initially have a high potential for metastatic disease and recurrence. Perrault et al. reported the same recurrence pattern in which all relapses were metastatic [17]. The radiological response was associated with a substantial impact on the outcome. The survival of patients with objective responses (CR and PR) was significantly better than those with MR, SD, and PD, which raises the importance of intensifying induction by CTH/RTH to achieve a better outcome. Mynarek M et al. concluded that the more intensified the chemotherapy induction (even without transplant), the better the remission status [15]. The 3-year OS and EFS for all cases were 46.7% and 44.4%, respectively. This survival was inferior compared to the survival reported by Liu et al. (5-year PFS and OS of 60.7 ± 6.6% and 61.0 ± 6.8%, respectively) [8]. In our study, the 3-year OS of M0 patients in the older group was 74% compared to 100% for the same group in the SJMB03 study; the high-dose chemotherapy used may be the cause of their better survival in older patients [9]. SIOP data concluded that the impact of HDCTH on survival is limited, but it was more evident in older patients [8]. The children oncology group (COG 99701) trial reported a much better 5-year OS and PFS of 81 ± 9% and 62 ± 11% [15]. The survival discrepancy between our study and the COG 99701 may be attributed to our center’s lack of chemotherapy dose intensity policy. In this study, the better infant survival in the M + ve group compared to the M0 group may be due to CSI administration to the only surviving patient. Mynarek et al. reported that 3/5 patients treated with focal RTH and transplant and 3/16 treated with CSI were cured in the younger age group [15]. RCT is needed to evaluate the real impact of mega therapy in pineoblastoma, especially in young patients. There is great need to classify pineoblastoma molecularly based on copy number, whole exome sequencing analysis to label patients with poor prognostic molecular groups (MYC, RB) who need intensified therapy, and those with excellent survival who harbored post-transcriptional regulators endonucleases mutations as DROSHA, DGCR8, and DICER1 with survival up to 100% [18]. The present study limitations were its retrospective design, relatively small number of patients, low number of patients with GTR, lack of molecular subtyping to detect patients with high risk for relapse, inability to offer transplant especially in infants due to long waiting list and lack of adoption of chemotherapy dose intensity policy .

## Conclusions

A multimodality approach is needed to treat this aggressive disease. Inadequate dose intensity affected our patients’ outcome negatively. A more aggressive approach using high-dose chemotherapy or CSI is required to improve infantile pineoblastoma’s dismal outcome. Focal radiotherapy is not an efficacious treatment in infants due to its high-metastatic potential. Molecular typing should be considered to label patients who need a more intensified approach and patients with favorable biology tumors who can be exploited to reduce treatment intensity.

## Data Availability

All data generated or analyzed during this study are included in this published article and its supplementary information files. We also agree that the copyright for my/our article will be transferred to journal of Egyptian National Cancer institute when it is accepted for publication.

## Abbreviations

CSI: Craniospinal irradiation
PBs: Pineoblastomas
CTH: Chemotherapy
RTH: Radiotherapy
HDCTH: High-dose chemotherapy
GTR: Gross total resection
NGGCT: Non-germinomatous germ cell tumor
V/P: Ventriculo peritoneal
OS: Overall survival
EFS: Event-free survival
CSF: Craniospinal fluid
NTR: Near-total resection
STR: Subtotal resection
CR: Complete response
PR: Partial response
MR: Minimal response
SD: Stable disease
PD: Progressive disease
PNETs: Primitive neuroectodermal tumors
RCT: Randomized controlled trial
COG: Children oncology group
